# The Expression of Wound-Healing-Related Histochemical and Immunohistochemical Stains at the Time of Salvage Isolated Neck Dissection—An Exploratory Study

**DOI:** 10.3390/cancers18182942

**Published:** 2026-09-11

**Authors:** Roel Henneman, Joyce Sanders, Ingrid Hofland, Olga Hamming-Vrieze, Alfons J. M. Balm, Erienne de Cuba

**Affiliations:** 1 Department of Head and Neck Oncology and Surgery, Netherlands Cancer Institute, 1006 BE Amsterdam, The Netherlands; 2 Department of Pathology, Netherlands Cancer Institute, 1006 BE Amsterdam, The Netherlands; 3 Core Facility Molecular Pathology and Biobanking, Netherlands Cancer Institute, 1006 BE Amsterdam, The Netherlands; 4 Department of Radiation Oncology, Netherlands Cancer Institute, 1006 BE Amsterdam, The Netherlands

**Keywords:** immunohistochemistry, complication, surgical site infection, radiotherapy, chemoradiotherapy, neck dissection, salvage therapy

## Abstract

**Introduction:** Salvage surgery is known for an increased surgical complication rate. However, currently available patient, tumor and therapy factors are not reliable enough to predict surgical site complications (SSCs). This exploratory study tests skin acquired by neck dissection for several histochemical and immunohistochemical stains to explore tissue changes related to wound healing. **Patients and methods:** From an existing database of 232 isolated neck dissections (NDs), 17 patients who underwent skin resection during ND were included—5 who underwent primary procedures and 12 who underwent salvage procedures. Skin specimens were tested for ten histochemical and immunohistochemical stains, with emphasis on the stages of wound healing: inflammation, proliferation and remodeling. **Results:** Three stains (CD-68, α-SMA, and Vimentin) showed significantly different expression between the primary surgical and salvage groups (respectively. *p* = 0.013, *p* = 0.044 and *p* = 0.015), without a clear pattern of decreased or increased expression. None of the ten stains showed a significant difference related to SSCs. **Conclusions:** The three significantly different staining patterns found in this exploratory study can all be linked to macrophage and (myo-)fibroblast transitions, possibly representing an imbalance of macrophage/stroma interplay. Future studies focusing on the role of macrophages in wound healing after salvage surgery are necessary.

## Simple Summary

Salvage surgery is known for an increased surgical complication rate. This study explores tissue differences after radiotherapy and chemoradiotherapy that can be related to impaired wound healing after salvage surgery. Ten routinely available immunohistochemical and histochemical stains were used with emphasis on the stages of wound healing: inflammation, proliferation, and remodeling. Skin specimens acquired by neck dissection from seventeen patients were tested. Three stains were found to be significantly different: CD-68, α-SMA, and Vimentin. However, they did not exhibit a clear pattern of decreased or increased expression. We were not able to find a difference in baseline vascularization levels. None of the ten stains showed a significant difference related to surgical site complications (SSCs). The above-mentioned three stains can be linked to macrophage and (myo-)fibroblast transitions. This outcome gives us direction to focus future research on macrophage transition and fibroblast activation.

## 1. Introduction

Salvage surgery of the head and neck area is known to have a higher risk of surgical site complications (SSCs) as compared to primary surgery. Quantifying this risk increase is difficult partly due to the variety and extent of surgical procedures—with or without resection of mucosal lining and adjacent neck dissection. One frequently performed procedure which lends itself to analysis because of its consistency is neck dissection (ND), performed as an *isolated* procedure with a clean surgical field—i.e., without contamination by saliva. NDs are well suited for a comparison of risk. When performed as an *isolated* primary procedure, there is a limited risk of surgical site complications [1]. However, when performed as a salvage procedure after radiotherapy or chemoradiotherapy, the risk of serious SSCs is higher, with incidences reported as high as 29% [2].

Another factor complicating analysis is that radiation therapy, either concurrent with chemotherapy or as sole treatment, has both early and late side effects, which can result in tissue alterations that can seriously disturb wound healing [3,4].

If disturbed wound healing is expected, one can try to prevent SSC by transferring well-vascularized muscular tissue to the wound in order to provide ‘healthy’ non-irradiated tissue as a basis for the wound healing process, for instance, by using a pedicled muscle flap intraoperatively [5,6]. However, this means a prolonged surgical procedure with additional costs and possible donor site morbidities. Knowing when such a flap is required is crucial to prevent overtreatment, as not each neck dissection after radiotherapy or chemoradiotherapy is complicated by SSCs.

To be able to predict which patients would benefit most from the use of the transfer of well-vascularized muscle tissue could therefore be helpful. Although impaired wound healing is strongly correlated with comorbidities and a variety of other patient-dependent factors affecting the general condition, these factors are not reliable enough to predict the outcome of wound healing in an individual case [7]. Ideally, if we had preoperative knowledge about both patient factors and tissue alterations related to wound healing, it would help us to predict more accurately which patient is at high risk for impaired wound healing. This should assist us in appropriately selecting patients for prophylactic use of pedicled flaps or free flap transfers.

This exploratory study was therefore intended to provide an initial insight into possible differences in tissue characteristics related to the subsequent phases of the wound healing process, in patients who underwent salvage *isolated* neck dissection with skin resection after radiotherapy or chemoradiotherapy, and with and without complication.

Although exploratory studies are sometimes also referred to as feasibility or pilot studies, the focus of this exploratory study is on discovery and hypothesis generation, rather than testing a study design.

## 2. Patients and Methods

We searched surgical and pathology reports for skin resection during neck dissection in a well-documented database of 219 consecutive patients (232 procedures), who had undergone isolated neck dissection (as primary treatment or as salvage post-radiotherapy or post-chemoradiotherapy) at the Netherlands Cancer Institute Antoni van Leeuwenhoek from 1997 until 2012 [7]. If skin resection was mentioned in one of these reports, the diagnostic HE-stained slides were reviewed.

The specimens from 17 neck-dissection procedures contained pathologically confirmed sufficient normal (i.e., non-tumorous) skin.

Informed consent was obtained from all patients before treatment, by stating that resected tissue is available for research purposes anonymously studying future options for personalized treatment. Approval of the Institutional Review Board was acquired, locally registered as CFMPB673.

The literature was searched for histochemical (HC) and immunohistochemical (IHC) staining previously used to study the characteristics of tissue involved in the different stages of healing, i.e., hemostasis, inflammation, proliferation and remodeling. Because of the exploratory setting and to enable future routine use, frequently used and readily available stains were selected in the first instance.

This selection process yielded the following ten stains: ALK-5A4 (anaplastic lymphoma kinase), Ki-67, CD-68, Elastica van Gieson, D2-40, alpha smooth muscle actin (α-SMA), Alcian Blue, Collagen IV, CD-34, and Vimentin. Their putative involvement in the wound healing process is summarized below and in Table 1.

• The 5A4 tests for the receptor of TGF (transforming growth factor) β type 1 [8]. While TGFβ 1 plays a role in normal wound healing, its expression has been found to be higher in cases of excessive fibrosis [9]. During hemostasis, TGFβ acts as a chemoattractant for monocytes, which are important to initiate the inflammation phase [10].
• CD-68 visualizes macrophages, activated to either *M1* or *M2*, which are essential for the inflammatory and the proliferative phase of wound healing, respectively [10]. Its adherence to the vascular intima and media makes it useful as a marker for vascular status [11]. CD-68 also detects fibroblasts [12].
• Vimentin staining detects activated fibroblasts [13], but also any monocytes and macrophages present during inflammation. We distinguished between fibroblasts and monocytes/macrophages by comparing all Vimentin-stained slides with slides stained with CD-68 [11] and hematoxylin–eosin (HE). Vimentin-deficient wounds show less fibroblast growth, which prevents the initiation of epithelial–mesenchymal transition (EMT) [14].
• CD-34, a known marker for hematopoietic stem cells, is also a marker for vascular endothelial progenitors and embryonic fibroblasts [15,16]. As a result of prior trauma (e.g., radiotherapy) to the epidermis, multipotent epithelial stem cells, partly CD34 positive, migrate to the site of injury.
• D2-40 detects expression of lymphatic endothelium [17].
• Alcian Blue indicates the presence of glycosaminoglycans, an important ingredient of the extracellular matrix (ECM) [18]. These affect the viscoelastic properties of the tissue by trapping water in the ECM [16].
• Ki-67 is used as a proliferation marker, highlighting the proliferative activity in the parabasal and basal cell layers in cases of skin damage repair [11,19].
• Elastica van Gieson staining indicates the presence of elastic fibers in connective tissue and also in the thickening of the vascular intima and media [20].
• α-SMA is induced by TGFβ 1 and expressed by myofibroblasts, which are present during wound healing and are involved in wound contraction. Comparing its expression between slides helps to identify differences in myofibroblast levels in underactive and overactive healing tissue relative to normal healing tissue [21,22,23].
• Collagen IV staining indicates the collagen levels present in the basal lamina and the vessels in the connective tissue. It also indicates vascular status [24,25,26,27].

### 2.1. Immunohistochemistry

Immunohistochemistry of the FFPE tumor samples was performed on a BenchMark Ultra autostainer (Ventana Medical Systems, Tucson, AZ, USA). Briefly, paraffin sections were cut at 3 μm, heated at 75 °C for 28 min and deparaffinized in the instrument with EZ prep solution (Ventana Medical Systems). Heat-induced antigen retrieval was carried out using Cell Conditioning 1 (CC1, Ventana Medical Systems) for 8 min at 95 °C (alpha-SMA), 32 min at 95 °C (CD68, D2-40, CD34, Vimentin), or 64 min at 95 °C (ALK, Ki67).

For Collagen IV enzymatic retrieval was carried out using Protease 1 (Ventana Medical Systems), 4 min at RT.

ALK was detected using clone 5A4 (1/40 dilution, 32 min at 37 °C, (LEICA, Deer Park, IL, USA)), Ki67 using clone MIB1 (1/100 dilution, 1 h at 37 °C, Agilent/DAKO, Santa Clara, CA, USA), CD68 using clone KP1 (1/10.000 dilution, 32 min at 37 °C, Agilent/DAKO), CD34 using clone QBEnd/10 (1/250 dilution, 32 min at 37 °C, NeoMarkers, Fremont, CA, USA), Podoplanin clone D2-40 (1/40 dilution, 32 min at 37 °C, Agilent/DAKO), alpha-SMA using clone 1A4 (1/4000 dilution, 32 min at 37 °C, Agilent/DAKO), Collagen IV using a cocktail (1/50 dilution, 32 min at 37 °C, NeoMarkers) and Vimentin, clone Vim3B4 (Agilent/DAKO) at 1/5000 dilution for 32 min at 37 °C.

For ALK and Vimentin, signal amplification was applied using the OptiView Amplification Kit (Ventana Medical Systems), 4 min (Vimentin) or 8 min (ALK).

Bound antibody was detected using the OptiView DAB Detection Kit (Ventana Medical Systems). Slides were counterstained with hematoxylin and Bluing Reagent (Ventana Medical Systems).

### 2.2. Histology

Elastica van Gieson and Alcian Blue staining of the FFPE tumor samples were performed manually.

For the Elastica van Gieson staining, slides were incubated with Lawson’s solution (10 min at 37 °C, Clin-Tech Ltd., Roterham, UK) followed by van Gieson solution (Acid Fuchsin (Bio-Optica Milano S.p.A, Milano, Italy) + Picric Acid (VWR Chemicals, Radnor, PA, USA), 5 min at RT.

For Alcian Blue, the slides were incubated with Alcian Blue (15 min at RT., Sigma-Aldrich, St. Louis, MO, SA) followed by Nuclear Fast Red (10 min at RT., Sigma-Aldrich). The slides were scanned with the Aperio AT2 scanner (Leica Biosystems Nussloch GmbH, Nußloch, Germany) and scored with the online platform Slidescore (www.slidescore.com).

### 2.3. Scoring

The skin of one patient (<70 years old, no tobacco or alcohol consumption, not underweight) who underwent primary surgical neck dissection without surgical site complications was used as a baseline reference. Of all the other specimens, expression of the HC and IHC stains was scored on a five-point scale compared to this ND, blinded to both initial therapy and clinical outcome (JS, RH).

Results were collected together with known patient and clinical characteristics from the existing database.

### 2.4. Statistical Analysis

To enable crosstab testing for the small numbers, expression outcomes were converted into a dichotomous variable. Staining patterns were divided into *normal* versus *decreased or increased* and, based on expected alteration by radiotherapy, into *reduced versus normal or increased*, or into *increased* versus *normal or decreased*. Testing was conducted using Fisher’s exact test, or exact if the number of observations was smaller than 5 in at least one of the evaluated subgroups. *p*-values were two-sided or one-sided when only an increase or decrease was tested.

All statistical analyses were performed using Statistical Package for the Social Science (SPSS) version 27.0 (SPSS Inc., Chicago, IL, USA).

## 3. Results

Pathology reports of 232 isolated neck dissections were screened. Skin tissue of sufficient quantity and quality for analysis was found in 17 patients, 15 males and 2 females. The seventeen included neck dissections were subdivided into five primary surgeries, one post-radiotherapy and eleven post-chemoradiotherapy. Radiotherapy was given in a total dosage of 70 Gray (N = 12), and concurrent chemotherapy consisted of administration of cisplatin (N = 11). The salvage procedures were grouped together. No significant difference in sex (*p* = 0.515) or age (*p* = 0.205) was seen between the primary surgical and salvage group; see Table 2 for all characteristics. Also, no significant difference was seen in type of ND performed (*p* = 0.261) or whether the sternocleidomastoid muscle was resected (*p* = 0.515). Use of a muscle flap (in all cases, the pedicled myofascial pectoralis major flap) was performed at the surgeon’s discretion in 20% (1/5) in the primary surgical group and in 67% (8/12) in the salvage group (*p* = 0.131). Although the length of surgery ranged from 48 to 350 min, no significant difference in median surgery time was seen between groups (*p* = 0.598).

Seven patients (41%) experienced a surgical site complication (SSC) which required active therapy (i.e., Clavien-Dindo (CD) > 1) (see Table 3). Wound infections were divided into superficial incisional and deep incisional, according to the Common Centers for Disease Control definitions of surgical site infection (SSI). Seven NDs were complicated by SSI, not significantly varying between both groups (*p* = 1.00).

In the primary ND group, one patient developed a seroma and one had necrosis of the skin around the incision. In the salvage group, in one patient a hemorrhage led to operative intervention (CD 3b). In addition, one patient had skin necrosis and one patient had wound dehiscence.

### Histopathology

Difference in degree of fibrosis between the primary surgical and salvage group was not found by use of ALK-5A4 staining. Both in the epithelium and in the stroma, no differences were seen (respectively, *p* = 0.294 and *p* = 1.00). An overview of HC/IHC staining in both the primary surgical and salvage group is shown in Table 4.

Expression of CD-68 in the dermis differed between these groups, *p* = 0.013. However, although the staining was abnormal after radiotherapy, the scoring showed either under- or overexpression without a clear trend toward either value (*p* = 0.106).

Also, Vimentin expression was abnormal in the salvage group as compared to the control group (*p* = 0.015), with both under- and overexpression.

When combining expression of HE, CD-68 and Vimentin to evaluate the quantity of fibroblasts, no significant differences were found.

There were no differences observed in the quantity of blood vessels and fibroblasts (highlighted by CD-34) and lymphatic structures (highlighted by D2-40) (*p* = 1.00 and *p* = 0.560, respectively).

Alcian Blue demonstrated varying staining patterns with no obvious normal pattern within both groups (*p* = 0.083).

The rate of proliferation by use of Ki-67 for also epithelium and stroma did not differ, *p* = 0.547 and *p* = 1.00. Elastica von Gieson staining demonstrated no difference between groups (*p* = 1.00); in only 2 salvage patients it was increased, while the other 15 patients showed normal expression.

Testing α-SMA for smooth muscle cells and activated fibroblasts showed a significant difference in staining in the salvage group (*p* = 0.044). However, no significant decrease or increase in α-SMA level was seen between both surgical groups (*p* = 0.128).

Collagen IV staining in both stroma and as an instrument to assess vascularization showed no difference between the groups (*p* = 0.588 and *p* = 0.516, respectively).

Table 5 shows the HC and IHC staining patterns grouped by the occurrence of surgical site complication (i.e., CD 1 versus CD grade > 1). For all different stains used, no significant different expressions were seen.

## 4. Discussion

This hypothesis-generating study was set up to investigate potential differences in staining patterns related to the process of wound healing, which can be influenced by previous radiotherapy or chemoradiotherapy.

It is a well-known clinical observation that the irradiated skin shows more fibrosis and is less well vascularized, though, for both characteristics, there is no simple histologic grading system [4]. Unfortunately, in this study, we were not able to unravel any unequivocal tissue alterations or immunohistochemical expression profiles that directly relate to the impairment of normal wound healing.

Macrophages play a key role in the normal wound healing process [10,28]. In the literature, studies report the macrophage-to-myofibroblast transition, identified by, respectively, CD-68 and α-SMA [29,30], with Vimentin acting as a mediator [31]. A low level of α-SMA could reflect fewer myofibroblasts, resulting in less collagen and thereby decreased ability for wound contraction [13]. A loss of Vimentin finally leads to a deficient growth of fibroblasts [14].

When comparing the specimens from the salvage surgery group to the primary surgery group, significant differences are seen in CD-68, α-SMA and Vimentin expressions (respectively, *p* = 0.013, *p* = 0.044 and *p* = 0.015), without a clear increase or decrease in staining activity. These findings suggest an imbalance in the tissue microenvironment of the irradiated patients at the start of wound healing mediated by macrophages, which could potentially lead to a prolonged inflammatory phase (with M1 macrophages) without reaching the next cascades in wound healing.

Also, NDs with a CDC > 1 did not show a different number of macrophages than the ones with CDC 1, which might indicate that quantity counts less than the condition of individual cells (Table 5).

Apart from the supposed disruption of the macrophage-to-myofibroblast transition, differences in the ability of regeneration of blood vessels after primary radiotherapy could be the cause of decreased wound healing capacity as well. Although CD-34 and collagen IV expression should give more insight into tissue vascularization, we were not able to find any significant correlation between the levels of expression and complications. However, from an angiogenesis point of view, Vimentin and α-SMA expression could shed light on the role of pericytes, which in the future could be a focus of further research in this specific clinical setting.

An attempt to combine expressions of both CD-68 and Vimentin in relation to HE, to distinguish from inflammation and to determine a microscopic degree of fibrosis, did not lead to a significant difference in the salvage group compared to the primary group nor in the group with CDC > 1 compared to the group with CDC < 1.

### Limitations

As this is an exploratory study, limitations are found in various aspects.

The main limiting factor for patient selection in this study was the limited availability of non-tumorous skin tissue. Skin resection is not common practice during neck dissection and is indicated in cases of invasion of tumor into the skin, thereby biasing the available skin specimens. Our interest was in tumor-negative skin. However, tumor-negative skin in salvage surgery specimens is scarce in pathology archives. A future prospective study could easily overcome this limitation. Furthermore, in a prospective setting, other relevant factors, such as various parameters of nutritional state of the patient, could also be noted and studied in greater detail.

Secondly, an important limitation seems to be the static aspect of this study. Skin was resected during surgery, which is the very start of the dynamic wound healing process.

The small numbers made it statistically challenging to find one-way correlations (i.e., either decrease or increase in expression) with radiotherapy and chemoradiotherapy. Also, the small numbers prevented us finding predictive factors of wound healing problems, with a bias in mind that in the salvage group a high percentage (67%) of pedicled muscle flaps were used to prevent wound healing disorders.

## 5. Conclusions

In conclusion, significantly different expressions of α-SMA, CD-68 and Vimentin were observed in the salvage surgery group. No difference in CD-34 was seen, suggesting that studying differences in vascularization in future research necessitates a different approach.

All these stains focus on macrophage and (myo-)fibroblast transition. The different expressions possibly represent an imbalance in the tissue microenvironment that affects macrophage function [28] and could be (though not fully understood) the result of previous radiotherapy or chemoradiotherapy.

### Future Perspectives

Despite the challenges faced, this exploratory study on HC/IHC markers—selected based on both cell biology knowledge and the literature—has shed some light on the complex matter of radiotherapy- and chemoradiotherapy-associated wound healing issues in neck dissection. Prediction of disturbed wound healing after salvage neck dissection remains difficult, but deserves further study from a tissue perspective.

Based on our current, albeit limited, data, we would propose a prospective study with assessment of the baseline presence of M1 and the transition to M2 macrophages and activation of fibroblasts together with the degree of vascularization at baseline. The latter could be achieved by focusing on pericytes, which could be performed by combining α-SMA and neuron-glial antigen 2 [32].

An increased sample size is necessary, preferably by using skin punch biopsies in both the high-dose radiation field and of the non-irradiated skin.

This approach helps us to study the preoperative identification of patients at risk for wound healing complications, allowing them to benefit from a prophylactic pedicled myofascial or free muscle flap transfer.

## Figures and Tables

**Table 1:** Histochemical and immunohistochemical (HC/IHC) stains and their putative involvement in the wound healing process.

Stain	Wound Healing Process					
	Hemostasis	Inflammation	Proliferation	Remodeling	Angiogenesis	Fibrosis
ALK-5A4	X					X
CD-68		X	X			X
Vimentin		X				X
CD-34					X	X
D2-40					X	
Alcian Blue				X		
Ki-67			X			
Elastica von Gieson				X	X	X
α-SMA			X	X		X
Collagen IV				X	X	

**Table 2:** Patient, tumor and therapy characteristics.

Characteristics				
	Total	Primary Surgery	Salvage Surgery	*p* Value
**Patients**				
Number of patients	17	5	12	
Sex (male/female)	15/2	4/1	11/1	0.515 ^
Age at surgery (year)				
median (range)	54 (28–83)	66 (46–83)	53.5 (28–65)	0.205 *
Tobacco consumption				1.000 #
- Never	1	0	1	
- Former	9	3	6	
- Active	7	2	5	
Alcohol consumption				1.000 #
- 0–2 IU/daily	6	2	4	
- 3–6 IU/daily	2	0	2	
- Abuse	9	3	6	
ASA				1.000 #
- 1	7	2	5	
- 2	9	3	6	
- 3	1	0	1	
BMI				0.381 #
- Underweight	2	0	2	
- Normal weight	11	3	8	
- Overweight	4	2	2	
**Tumor**				
Primary tumor				0.195 #
- Oral cavity	3	1	2	
- Oropharynx	10	2	8	
- Hypopharynx	2	0	2	
- Larynx	1	1	0	
- Other	1	1	0	
Nodal stage				1.000 #
- N1	2	1	1	
- N2	7	1	6	
- N3	8	3	5	
**Therapy**				
Type of ND				
- *(Super)* selective	4	0	4	0.261 ^
- (Modified) radical	13	5	8	
Resection SCM muscle				
- No	3	0	3	0.515 ^
- Yes	14	5	9	
Extracapsular spread				
- No	8	1	7	0.294 ^
- Yes	9	4	5	
Primary pedicled muscle flap transplantation				
- No	8	4	4	0.131 ^
- Yes	9	1	8	
Length of surgery (minutes)				
- median (range)	225 (48–350)	225 (135–328)	215 (48–350)	0.598 *

ASA = American Society of Anesthesiologist scale, BMI = body mass index, ND = neck dissection, SCM = sternocleidomastoid muscle; ^ = Fisher’s exact test, # = linear by linear test, * = Mann–Whitney U-test.

**Table 3:** Surgical site complications.

Surgical Site Complications				
	Total	Primary Surgery	Salvage Surgery	*p* Value
Clavien-Dindo				
- 1	10	3	7	1.00 ^
- >1 (therapy required)	7	2	5	
Surgical Site Infection (SSI)				
- No	10	3	7	
- Yes	7	2	5	1.00 ^
Type of SSI				
- Superficial	2	1	1	
- Deep	5	1	4	1.00 ^
Hemorrhage				
- No	16	5	11	
- Yes	1	0	1	1.00 ^
Hematoma				
- No	17	5	12	
- Yes	0	0	0	-
Seroma				
- No	16	4	12	
- Yes	1	1	0	0.294 ^
Skin necrosis				
- No	15	4	11	
- Yes	2	1	1	0.515 ^
Wound dehiscence				
- No	16	5	11	
- Yes	1	0	1	1.00 ^
Chyle leakage				
- No	17	5	12	
- Yes	0	0	0	-
Exposed carotid artery				
- No	17	5	12	
- Yes	0	0	0	-

^ = Fisher’s exact test; cumulative numbers of SSC exceed total complicated neck dissections, due to the possibility of >1 SSC per ND.

**Table 4:** Immunohistochemical (IHC) staining patterns between surgical groups.

	Primary Surgery		Salvage Surgery		
Marker	Expression				*p* Value
ALK-5A4	Normal	Deviant	Normal	Deviant	
	1	4	4	8	1.00 ^
	Reduced	Not reduced	Reduced	Not reduced	
	3	2	10	2	0.330 ^*
	Increased	Not increased	Increased	Not increased	
	2	3	6	6	0.563 ^*
CD-68	Normal	Deviant	Normal	Deviant	
	4	1	1	10	0.013 ^
	Increased	Not increased	Increased	Not increased	
	0	5	5	6	0.106 ^*
	Decreased	Not decreased	Decreased	Not decreased	
	4	1	6	5	0.346 ^*
Vimentin	Normal	Deviant	Normal	Deviant	
	3	2	0	12	0.015 ^
	Reduced	Not reduced	Reduced	Not reduced	
	1	4	6	6	0.278 ^*
CD-34	Normal	Deviant	Normal	Deviant	
	3	2	7	5	1.00 ^
D2-40	Normal	Deviant	Normal	Deviant	
	2	3	2	8	0.560 ^
	Reduced	Not reduced	Reduced	Not reduced	
	3	2	8	2	0.407 ^*
Alcian Blue	Normal	Deviant	Normal	Deviant	
	2	3	0	11	0.083 ^
	Reduced	Not reduced	Reduced	Not reduced	
	0	4	0	11	-
Ki-67	Normal	Deviant	Normal	Deviant	
	2	3	2	9	0.547 ^
	Reduced	Not reduced	Reduced	Not reduced	
	3	2	9	2	0.365 ^*
Elastica von Gieson	Normal	Deviant	Normal	Deviant	
	4	0	9	2	1.00^
	Increased	Not increased	Increased	Not increased	
	0	4	2	9	0.524^*
α-SMA	Normal	Deviant	Normal	Deviant	
	5	0	5	7	0.044 ^
	Reduced	Not reduced	Reduced	Not reduced	
	0	5	5	7	0.128 ^*
Collagen IV	Increased	Normal	Increased	Normal	
	4	1	6	5	0.588 ^
- blood vessel	Reduced	Not reduced	Reduced	Not reduced	
	3	2	8	3	0.516 ^*
Fibrosis (CD-68 + Vimentin + H&E †)	No	Increased	No	Increased	
	3	2	5	7	0.437 ^*

^ = Fisher’s exact test, * = one-sided test, † = hematoxylin and eosin.

**Table 5:** Histochemical and immunohistochemical (HC/IHC) staining patterns for surgical site complication defined by Clavien-Dindo grade 1 and >1.

	Clavien-Dindo Grade 1		Clavien-Dindo Grade > 1		
Marker	Expression				*p* Value
ALK-5A4	Normal	Deviant	Normal	Deviant	
	4	6	1	6	0.338 ^
	Increased	Not increased	Increased	Not increased	
	6	4	2	5	0.218 ^*
CD-68	Normal	Deviant	Normal	Deviant	
	4	5	1	6	0.308 ^
	Increased	Not increased	Increased	Not increased	
	2	7	3	4	0.365 ^*
Vimentin	Normal	Deviant	Normal	Deviant	
	1	9	5	2	0.537 ^
	Reduced	Not reduced	Reduced	Not reduced	
	5	5	2	5	0.354 ^*
CD-34	Normal	Deviant	Normal	Deviant	
	7	3	3	4	0.350 ^
D2-40	Normal	Deviant	Normal	Deviant	
	2	7	2	4	1.00 ^
	Reduced	Not reduced	Reduced	Not reduced	
	7	2	4	2	0.538 ^*
Alcian Blue	Normal	Deviant	Normal	Deviant	
	2	8	0	6	0.500 ^
	Reduced	Not reduced	Reduced	Not reduced	
	0	10	0	6	-
Ki-67	Normal	Deviant	Normal	Deviant	
	2	7	2	5	1.00 ^
	Reduced	Not reduced	Reduced	Not reduced	
	7	2	5	2	0.608 ^*
Elastica von Gieson	Normal	Deviant	Normal	Deviant	
	8	1	5	1	1.00 ^
	Increased	Not increased	Increased	Not increased	
	1	8	1	5	0.657 ^*
α-SMA	Normal	Deviant	Normal	Deviant	
	7	3	3	4	0.350 ^
	Reduced	Not reduced	Reduced	Not reduced	
	3	7	2	5	0.686 ^*
Collagen IV	Increased	Normal	Increased	Normal	
	4	6	2	4	0.608 ^*
- blood vessel	Reduced	Not reduced	Reduced	Not reduced	
	7	3	4	2	0.654 ^*
Fibrosis (CD-68 + Vimentin + H&E †)	No	Increased	No	Increased	
	5	5	3	4	0.581 ^*

^ = Fisher’s exact test, * = one-sided test, † = hematoxylin and eosin.

## Data Availability

The data presented in this study are available on request from the corresponding author if permitted by privacy and legal restrictions.
